# Association between mortality and age among mechanically ventilated COVID-19 patients: a Japanese nationwide COVID-19 database study

**DOI:** 10.1186/s13613-021-00959-6

**Published:** 2021-12-11

**Authors:** Chie Tanaka, Takashi Tagami, Fumihiko Nakayama, Saori Kudo, Akiko Takehara, Reo Fukuda, Junya Kaneko, Yoshito Ishiki, Shin Sato, Ami Shibata, Masamune Kuno, Kyoko Unemoto, Masayuki Hojo, Tetsuya Mizoue, Yusuke Asai, Setsuko Suzuki, Norio Ohmagari

**Affiliations:** 1 Department of Emergency and Critical Care Medicine, Nippon Medical School Tama Nagayama Hospital, Tama-shi, Tokyo 2068512 Japan; 2 Department of Emergency and Critical Care Medicine, Nippon Medical School Musashikosugi Hospital, 1-396 Kosugimachi, Nakahara-ku, Kawasaki, Kanagawa 211-8533 Japan; 3 Department of Clinical Epidemiology and Health Economics, School of Public Health, The University of Tokyo, Bunkyo, Tokyo 1138654 Japan; 4 Disease Control and Prevention Center, National Center for Global Health and Medicine, Shinjuku, Tokyo 1628655 Japan; 5 AMR Clinical Reference Center, National Center for Global Health and Medicine, Shinjuku, Tokyo 1628655 Japan; 6 Department of Epidemiology and Prevention, Center for Clinical Sciences, National Center for Global Health and Medicine, Shinjuku, Tokyo 1628655 Japan; 7 Department of Respiratory Medicine, National Center for Global Health and Medicine, Shinjuku, Tokyo 1628655 Japan

**Keywords:** COVID-19, Age, Mechanical ventilation, Mortality

## Abstract

**Background:**

Only a few studies have reported the association between age and mortality in COVID-19 patients who require invasive mechanical ventilation (IMV). We aimed to evaluate the effect of age on COVID-19-related mortality among patients undergoing IMV therapy.

**Methods:**

This cohort study was conducted using the COVID-19 Registry Japan database, a nationwide multi-centre study of hospitalized patients with laboratory-confirmed COVID-19. Of all 33,808 cases registered between 1 January 2020 to 28 February 2021, we analysed 1555 patients who had undergone IMV. We evaluated mortality rates between age groups using multivariable regression analysis after adjusting for known potential components, such as within-hospital clustering, comorbidities, steroid use, medication for COVID-19, and vital signs on admission, using generalized estimation equation.

**Results:**

By age group, the mortality rates in the IMV group were 8.6%, 20.7%, 34.9%, 49.7% and 83.3% for patients in their 50s, 60s, 70s, 80s, and 90s, respectively. Multivariable analysis showed that compared with those for patients aged < 60 years, the odds ratios (95% confidence interval) of death were 2.6 (1.6–4.1), 6.9 (4.2–11.3), 13.2 (7.2–24.1), 92.6 (16.7–515.0) for patients in their 60s, 70s, 80s, and 90s, respectively.

**Conclusions:**

In this cohort study, age had a great effect on mortality in COVID-19 patients undergoing IMV, after adjusting for variables independently associated with mortality. This study suggested that age was associated with higher mortality and that preventing progression to severe COVID-19 in elderly patients may be a great public health issue.

**Supplementary Information:**

The online version contains supplementary material available at 10.1186/s13613-021-00959-6.

## Background

In December 2019, the first case of the novel coronavirus disease 2019 (COVID-19), caused by severe acute respiratory syndrome coronavirus 2 (SARS-CoV-2), was reported in Wuhan, China [1]. The first COVID-19 case in Japan was reported on 16 January, 2020, and the World Health Organization (WHO) declared COVID-19 a pandemic on 11 March 2020 [2, 3]. Currently, the clinical spectrum of COVID-19 ranges from mild to severe, and the mortality rates of patients with severe cases undergoing invasive mechanical ventilation (IMV) are reportedly high [4–6]. Several studies have identified older age as an independent prognostic factor for mortality [4, 7–13]; however, limited information is available on the relationship between mortality and critically ill patients on IMV, stratified by age group [6, 7].

In Japan, people aged > 65 years accounted for 28.7% of the total population in 2020, and the number of frail elderly individuals has been increasing with advancing age. Moreover, Japan was confronted with a shortage of beds, staff members, and ventilators during the peak of the COVID-19 pandemic. Therefore, the association between age and mortality is an important clinical issue, especially in an aging society, in deciding whether to perform IMV therapy for older adults.

We aimed to investigate the patterns of in-hospital mortality among critically ill patients with COVID-19 who required IMV, by age group, in Japan, while adjusting for other factors related to mortality.

## Methods

### Ethics approval

The present study adhered to the principles of the Declaration of Helsinki and was approved by the ethics committee of the Nippon Medical School Tama Nagayama Hospital. As anonymous data were analysed, the requirement for informed consent was waived.

### Settings

The basic policies of the Ministry of Health, Labour, and Welfare of Japan for the treatment of COVID-19 patients are as follows: all patients with a positive SARS-CoV-2 test result, diagnosed with COVID-19 are admitted to the hospital, while some asymptomatic COVID-19 patients or COVID-19 patients who do not require medical care are isolated either at home or at a designated hotel.

The health system in Japan ensures that the quality of medication use is homogenized, owing to the Japanese universal health insurance coverage system. The insurance system warrants a health check-up at any hospital of the patient’s choice, and that the patients are transported to the nearest hospital in an ambulance. All patients fundamentally receive the same healthcare services provided by the health insurance system in all hospitals, although the medical staff decide the treatment strategy considering the patients’ age, activity of daily living, medical history, and patients’ or their family’s intentions when the patients are critically ill and require intensive care.

### Study design

We conducted an observational cohort study using the COVID-19 Registry Japan (COVIREGI-JP) database, a nationwide, multi-centre database created by the National Centre for Global Health and Medicine [14]. The COVIREGI-JP database contains data of hospitalized patients with laboratory-confirmed COVID-19 who were admitted after 1 January 2020, from 925 participating hospitals throughout Japan. These records include information about the patients’ age, sex, body mass index (BMI), comorbidities, cause of infection, symptoms, vital signs on admission day, vital signs during hospitalization, treatments, results of laboratory tests, drugs, complications, and outcome at discharge. The follow-up ends at the patients’ discharge or death. The study data were collected and managed using REDCap (Research Electronic Data Capture), a secure, web-based data capture application hosted at the Joint Centre for Researchers, Associates, and Clinicians data centre of the National Centre for Global Health and Medicine. Data from the COVIREGI-JP database, of the National Centre for Global Health and Medicine, were used for this study with permission.

### Participants

The present study included all COVID-19 patients who were admitted to a hospital and required IMV and were registered in the COVIREGI-JP database from 1 January 2020 to 28 February 2021.

### Outcome measures

The primary outcome was all-cause in-hospital mortality. The secondary outcomes were complications during hospitalization, tracheostomy at discharge, and oxygen therapy at discharge. Complications included bacterial pneumonia, acute respiratory distress syndrome (ARDS), meningitis, ventricular fibrillation, deep venous thrombosis, and pulmonary embolism.

### Definition

We considered the specified time course because the spread and treatment strategy for COVID-19 have been changing drastically over time. Accordingly, we classified the study duration into three periods, in accordance with the epidemic trends of COVID-19 in Japan: the first wave from 1 January 2020 to 31 May 2020, the second wave from 1 June 2020 to 30 September 2020, and the third wave occurred after 1 October 2020.

The actual diagnosis of ARDS was made by each participating doctor in charge in the clinical setting based on the Berlin definition 2012 [15], and the definitions of some variables using logistic regression analysis (comorbidity, immunosuppression, drug administration for COVID-19, and drug administration for coagulopathy) are shown in Additional file 1: Table S1.

### Statistical analysis

We stratified the records into 10 each by age group (in 10-year increments). Continuous values were expressed as mean (standard deviation), and categorical values were expressed as numbers (%). Since this was an observational study, values were compared using standardized difference [16]. Next, we contrasted survivors with non-survivors in the study group based on the characteristics, treatments, medications, and complications. Further, we performed multiple imputation to decrease the bias caused by incomplete data; each missing value was replaced with a set of five substitute plausible values [17, 18]. Models were constructed for each imputed dataset, and a single model was created by statistical inference with the results of the five imputed datasets. We performed multiple imputations of covariates via fully conditional specification, including all variables listed in Table 4 and outcomes. Then, we evaluated mortality between age groups using a multivariable regression analysis after adjusting for known potential components and for within-hospital clustering using generalized estimation equation. Treatments and patient care vary between hospitals, even though treatment guidelines for COVID-19 are followed [19, 20]. Therefore, we considered clustering effect within hospital groups. We selected variables independently associated with COVID-19 mortality for logistic regression analysis, referring to previous studies for clinically important factors [21, 22], and the variables were as follows: sex, steroid use, drug administration for COVID-19, the admission date, BMI (< 30 kg/m^2^, ≥ 30 kg/m^2^), fever (< 38 °C,  ≥ 38 °C), SpO2 < 90%, respiratory rate ≥ 30, Systolic blood pressure ≤ 80 mmHg, comorbidities, immunosuppression status, medication use for coagulopathy, ARDS, and days from symptom onset to IMV [4, 7, 8, 10–13, 23].

Statistical significance was set at *p* < 0.05. All data were analysed using IBM SPSS Statistics version 27 (IBM Corp., Armonk, NY, USA).

## Results

### Patient characteristics, vital signs, and symptoms at admission

**Fig. 1:**
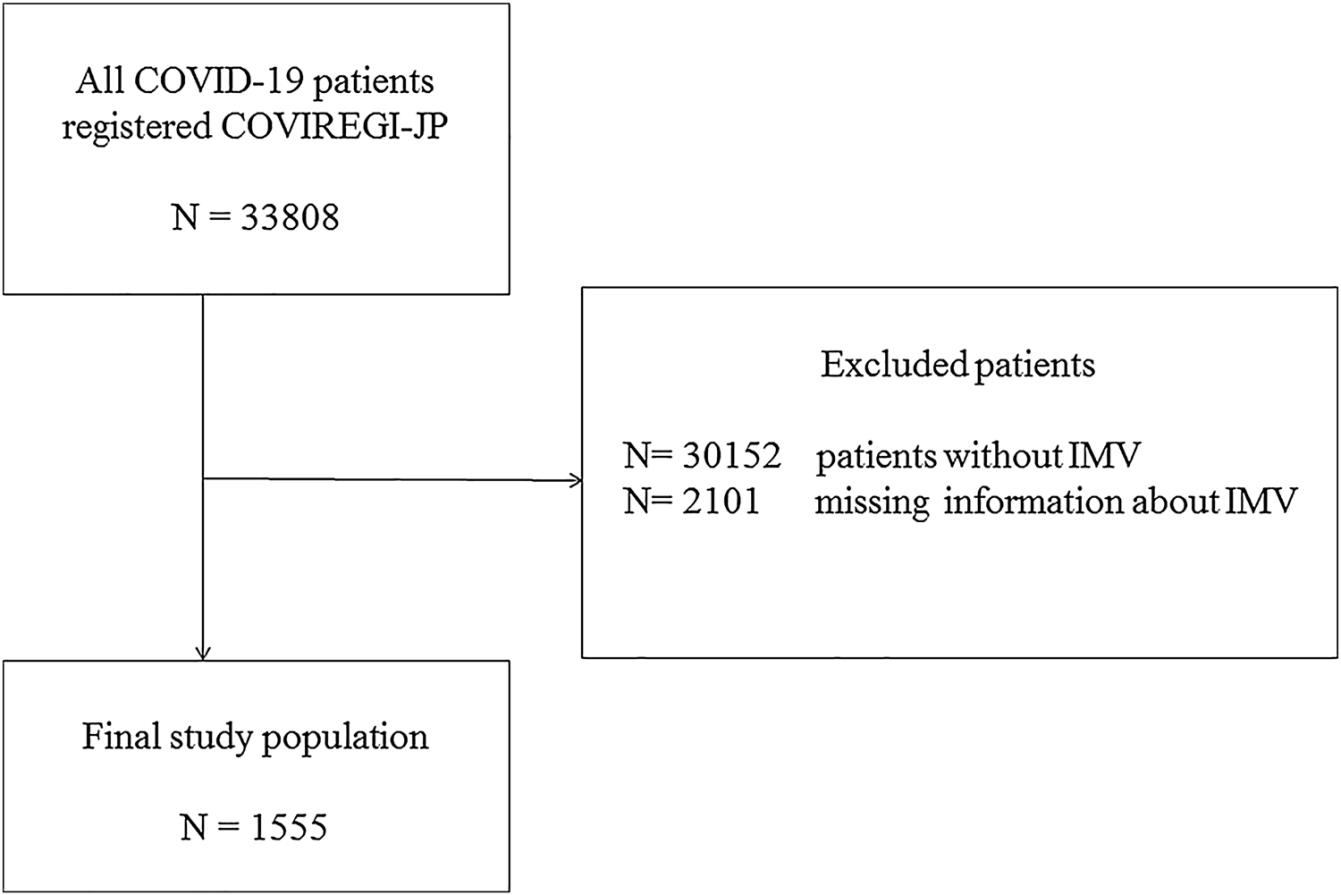
Patient selection flowchart

Of the data obtained from 33,808 cases, the final study population was 1555 (Fig. 1). The mean (standard deviation) age was 64.3 years (12.3) in the survivor group and 73.4 years (9.9) in the non-survivor group. There were 904 (80.3%) males in the survivor group and 306 (76.3%) in the non-survivor group.

The number of patients who had comorbidities and were immunosuppressed was 842 (74.6%) and 32 (2.9%), respectively, in the survivor group, and 336 (83.8%) and 23 (6.0%), respectively, in the non-survivor group. Fever (over 38 °C at admission) was reported in 369 (32.9%) patients in the survivor group and 110 (27.7%) in the non-survivor group. SpO_2_ under 90% at admission was observed in 163 (15.1%) patients in the survivor group and 71 (18.2%) in the non-survivor group. The number of patients with symptoms at admission was 1,088 (98.7%) in the survivor group and 378 (97.7%) in the non-survivor group (Table 1). More than 10% of the data were missing for BMI, smoking history, drinking history, and complication of deep vein thrombosis (Table 1).

**Table 1:** Demographic and clinical characteristics of patients with coronavirus disease 2019 under invasive mechanical ventilation therapy

Variables	Survivor (*n* = 1128)		Non-survivor (*n* = 401)		Standardized difference, %
Age, years	64.3 (12.3)		73.4 (9.9)		− 81.5
Male	904/1126 (80.3)		306/401 (76.3)		9.72
Body mass index ≥ 30	157/940 (16.7)		40/314 (12.7)		11.31
Admission date					
3rd wave (October 1st–)	484/1126 (43.0)		175/401 (43.6)		− 1.21
2nd wave (June 1st, 2020–September 30, 2020)	258/1126 (22.9)		71/401 (17.7)		12.95
1st wave (January 26, 2020–May 31, 2020)	384/1126 (34.1)		155/401 (38.7)		− 9.57
Race					
Japanese	1091/1120 (97.4)		391/395 (99.0)		− 12.06
Smoking history	527/866 (60.9)		177/288 (61.5)		− 1.23
Drinking history	432/709 (60.9)		121/229 (52.8)		16.41
Comorbidity	842/1128 (74.6)		336/401 (83.8)		− 22.81
Hypertension	552/1128 (48.9)		193/401 (48.1)		1.60
Diabetes	389/1128 (34.5)		158/401 (39.4)		− 10.16
Hyperlipidemia	256/1128 (22.7)		97/401 (24.2)		− 3.54
Cerebrovascular disease	97/1128 (8.6)		54/401 (13.5)		15.68
COPD	71/1128 (6.3)		30/401 (7.5)		− 4.74
Bronchial asthma	63/1128 (5.6)		19/401 (4.7)		4.07
Solid tumor	50/1128 (4.4)		32/401 (8.0)		− 14.97
Liver disease	46/1128 (4.1)		22/401 (5.5)		− 6.55
Moderate-to-severe chronic kidney disease	41/1128 (3.6)		29/401 (7.2)		− 15.98
Ischemic heart disease	41/1128 (3.6)		26/401 (6.5)		− 13.27
Congestive heart failure	35/1128 (3.1)		25/401 (6.2)		− 14.76
Major neurocognitive disorder	28/1128 (2.5)		28/401 (8.3)		− 25.88
Chronic lung disease excluding COPD	28/1128 (2.5)		26/401 (6.5)		− 19.39
Hemodialysis before admission	28/1128 (3.1)		22/401 (5.5)		− 11.85
Collagen disease	19/1128 (1.7)		11/401 (2.7)		− 6.82
Metastatic solid tumor	7/1128 (0.6)		7/401 (1.7)		− 10.33
Lymphoma	7/1128 (0.6)		5/401 (1.2)		− 6.36
Leukemia	0/1128 (0)		2/401 (0.5)		− 10.33
Immunosuppression	32/1091 (2.9)		23/384 (6.0)		0.01
Vital signs on admission					
AVPU scale A (Alert)	846/1021 (82.8)		281/366 (76.8)		14.99
Fever (≥ 38 °C)	369/1121 (32.9)		110/397 (27.7)		11.33
Respiratory rate ≥ 30 breaths/ minute	168/1011 (16.6)		61/359 (17.0)		− 1.07
SpO2 < 90%	163/1114 (15.1)		71/391 (18.2)		− 8.33
Systolic blood pressure ≤ 80 mmHg	20/1112 (1.8)		3/396 (0.8)		8.84
Symptoms at admission	1088/1102 (98.7)		378/387 (97.7)		7.53

Analysis based on records from the COVID-19 Registry Japan. Data given as number of positive observations/total number of observations (percentage) or as mean (standard deviation). For each variable, the number of missing observations can be obtained as the difference between the total number of patients in each phase and the total number of observations

*COPD* chronic obstructive pulmonary disease, *SD* standardized difference, *IMV* invasive mechanical ventilation

For continuous variables, the standardized difference (d) is defined as follows:

d = ($$\overline{\chi }$$_survivor_ – $$\overline{\chi }$$_non-survivor_)/$$\sqrt{{(s}^{2}\mathrm{ survivor }+{s}^{2 }\mathrm{non}-\mathrm{survivor})/2}$$

For dichotomous variables, the standardized difference is defined as follows:

d = ($$\widehat{p}$$_survivor_ –$$\widehat{p}$$_non-survivor_)/$$\sqrt{\left\{\widehat{p} \mathrm{survivor }\left(1-\widehat{p} \mathrm{non}-\mathrm{survivor} \right)+\widehat{p} \mathrm{survivor }\left(1-\widehat{p} \mathrm{non}-\mathrm{survivor}\right)\right\}/2}$$

$\overline{\chi }$: mean, s: standard deviation, $\widehat{p}$: proportion

### Mortality by age group

**Fig. 2:**
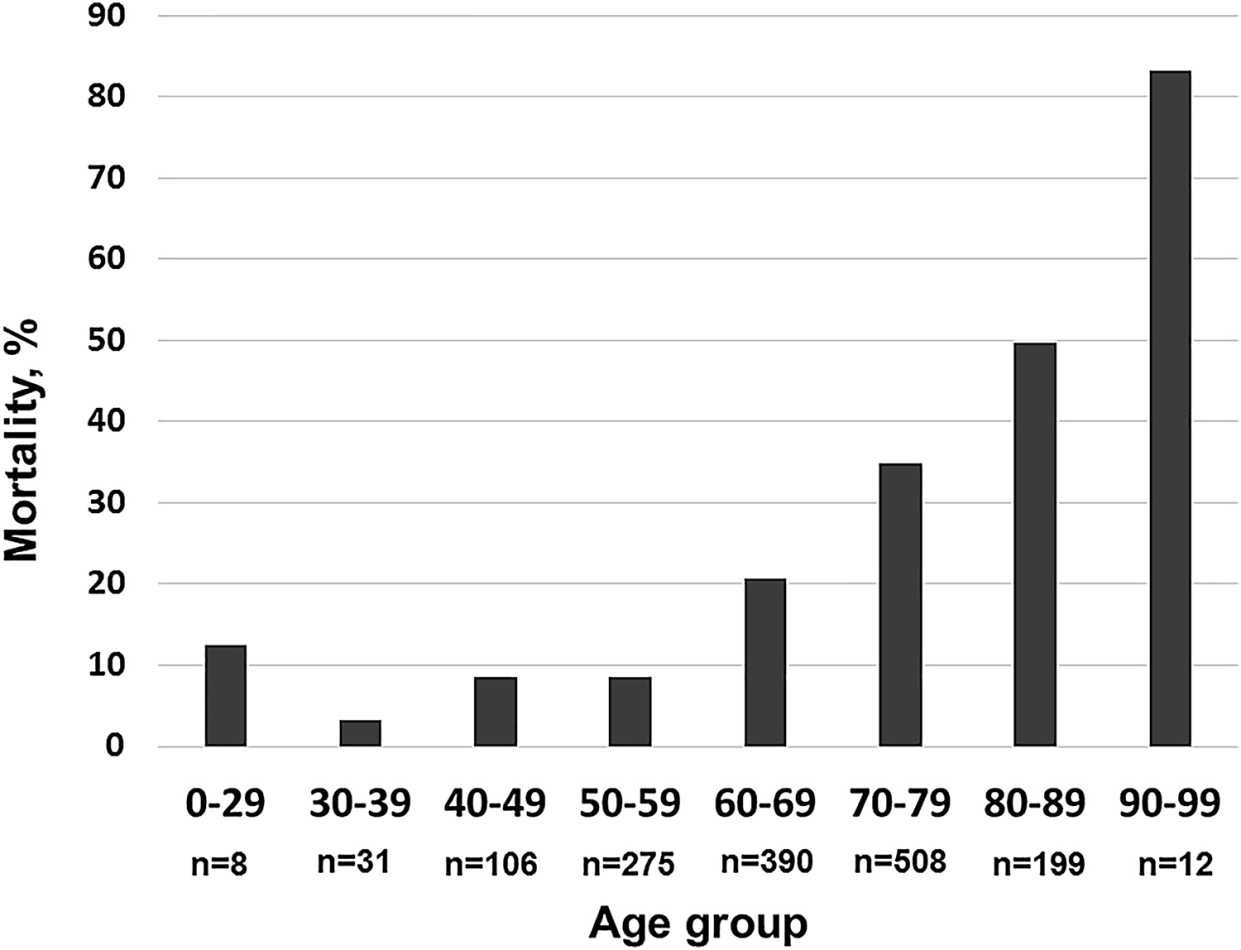
Mortality by age group

The in-hospital mortality rate was 26.3% (401/1529). When assessed by the age groups, the mortality rate was 8.6% (24/278), 20.7% (80/387), 34.9% (177/507), 49.7% (99/199), and 83.3% (10/12) for patients in their 50 s, 60 s, 70 s, 80 s, and 90 s, respectively (Fig. 2).

### Treatments and complications

The mean (standard deviation) number of days from symptom onset to IMV therapy was 8.8 days (13.3) for patients who survived and 8.7 days (6.2) for those who died. The mean (standard deviation) duration of IMV therapy was 10.6 days (13.3) for patients who survived and 17.4 days (24.1) for those who died. The percentage of patients who were administered medication for COVID-19 was 89.8% (1008/1122) among survivors and 89.1% (353/396) among non-survivors; 69.6% (767/1102) and 72.2% (286/396) were administered steroids among survivors and non-survivors, respectively (Table 2). The prevalence of severe ARDS was 20.4% (212/1039) in the survivor group and 63.3% (217/343) in the non-survivor group. The number of patients who underwent tracheotomy and oxygen therapy at discharge were 116 (10.3%) and 563 (49.9%), respectively (Table 3).

**Table 2:** Treatments of patients with coronavirus disease 2019 under invasive mechanical ventilation therapy

Variables	Survivor (*n* = 1128)		Non-survivor (*n* = 401)		Standardized difference, %
Drug administration for COVID-19	1008/1122 (89.8)		353/396 (89.1)		2.28
Favipiravir	545/993 (54.9)		200/349 (57.3)		− 4.84
Remdesivir	437/987 (44.3)		127/347 (36.6)		15.74
Ciclesonide	231/991 (23.3)		73/347 (21.0)		5.54
Nafamostat	156/964 (16.2)		66/337 (19.6)		− 8.88
Tocilizumab	123/964 (12.8)		37/337 (11.0)		5.56
Hydroxychloroquine	56/991 (5.7)		26/348 (7.5)		− 7.25
Lopinavir and ritonavir	50/991 (5.0)		23/347 (6.6)		− 6.85
Ivermectin	7/977 (0.7)		2/344 (0.6)		1.24
Interferon	3/1007 (0.3)		2/355 (0.6)		− 4.48
Baricitinib	0/978 (0)		1/344 (0.3)		− 7.76
Antibiotics	832/1103 (75.4)		336/393 (85.5)		− 25.68
Antifungal agent	60/1105 (5.4)		60/390 (15.4)		− 33.21
Neuraminidase inhibitor	12/1099 (1.1)		10/389 (2.6)		− 11.15
Steroid (excluding ciclesonide)	767/1102 (69.6)		286/396 (72.2)		− 5.73
Drug administration for coagulopathy	695/1066 (65.2)		252/376 (67.0)		− 3.80
Anticoagulant agents	655/1126 (58.2)		239/401 (59.6)		− 2.85
Antiplatelet agents	109/1066 (10.2)		47/376 (12.6)		− 7.56
Thrombolytic agents	12/1068 (1.1)		5/376 (1.3)		− 1.84
Plasmapheresis	8/1124 (0.7)		6/399 (1.5)		− 7.68
Immunoglobulin	57/1124 (5.1)		43/397 (10.8)		− 21.19
Vasopressor/inotropic support	430/1116 (38.5)		259/395 (65.6)		− 56.36
Renal replacement therapy	93/1117 (8.3)		120/399 (30.1)		− 57.60
Prone positioning	344/1109 (31.0)		142/397 (35.8)		− 10.19
High-flow oxygen device use	222/1120 (19.8)		88/398 (22.1)		− 5.65
Noninvasive positive pressure ventilation	147/1124 (13.1)		42/398 (10.6)		7.74
Duration of symptom onset to IMV, days	8.8 (13.3)		8.7 (6.2)		0.96
Duration of IMV, days	10.6 (13.3)		17.4 (24.1)		− 34.9
Re-intubation	43/1094 (3.9)		26/396 (6.6)		− 12.13
Nitric oxide inhalation	17/1118 (1.5)		12/399 (3.0)		− 10.13
Neuromuscular blocking agent	561/1067 (52.6)		184/374 (49.2)		6.81
Tracheotomy	172/1119 (15.4)		84/399 (21.1)		− 14.80
Extracorporeal membrane oxygenation	106/1127 (9.4)		45/401 (11.2)		− 5.92

Analysis based on records from the COVID-19 Registry Japan. Data given as number of positive observations/total number of observations (percentage) or as mean (standard deviation). For each variable, the number of missing observations can be obtained as the difference between the total number of patients in each phase and the total number of observations

*IMV* invasive mechanical ventilation, *SD* standardized difference

For continuous variables, the standardized difference (*d*) is defined as follows:

d = ($$\overline{\chi }$$_survivor_ – $$\overline{\chi }$$_non-survivor_)/$$\sqrt{{(s}^{2}\mathrm{ survivor }+{s}^{2 }\mathrm{non}-\mathrm{survivor})/2}$$

For dichotomous variables, the standardized difference is defined as follows:

d = ($$\widehat{p}$$_survivor_ –$$\widehat{p}$$_non-survivor_)/$$\sqrt{\left\{\widehat{p} \mathrm{survivor }\left(1-\widehat{p} \mathrm{non}-\mathrm{survivor} \right)+\widehat{p} \mathrm{survivor }\left(1-\widehat{p} \mathrm{non}-\mathrm{survivor}\right)\right\}/2}$$

$\overline{\chi }$: mean, s: standard deviation, $\widehat{p}$: proportion

**Table 3:** Outcomes of patients with coronavirus disease 2019 under invasive mechanical ventilation therapy

Variables	Survivor (*n* = 1128)		Non-survivor (*n* = 401)		Standardize*d* difference, %
Complications					
Viral pneumonia (excluding COVID-19)	39/1057 (3.5)		24/358 (6.7)		− 14.58
Bacterial pneumonia	290/1051 (27.6)		180/360 (50.0)		− 47.23
Acute respiratory distress syndrome					
None	460/1039 (44.3)		88/343 (25.7)		39.76
Mild	85/1039 (8.2)		6/343 (1.7)		30.31
Moderate	282/1039 (27.1)		32/343 (9.3)		47.41
Severe	212/1039 (20.4)		217/343 (63.3)		− 96.57
Pleural effusion	144/1082 (13.3)		98/371 (26.4)		− 33.29
Bacteremia	79/1093 (7.2)		71/371 (19.1)		− 35.77
Deep vein thrombosis	61/995 (6.1)		19/326 (5.8)		1.27
Pneumothorax	33/1093 (3.0)		51/380 (13.4)		− 38.61
Hemoptysis	31/1050 (3.0)		34/361 (9.4)		− 26.78
Ventricular defibrillation, ventricular tachycardia	28/1097 (2.6)		30/376 (8.0)		− 24.28
Gastrointestinal bleeding	23/1094 (2.1)		46/379 (12.1)		− 39.70
Seizures	21/1101 (1.9)		5/381 (1.3)		4.78
Cerebral infarction, cerebral hemorrhage	20/1104 (1.8)		21/372 (5.6)		− 20.23
Pulmonary embolism	20/1022 (2.0)		14/335 (4.2)		− 12.72
Ischemic heart disease	18/1098 (1.6)		8/374 (2.1)		− 3.71
Myocarditis, pericarditis, cardiomyopathy	10/1100 (0.9)		4/373 (1.1)		− 2.01
Meningitis, encephalitis	7/1075 (0.7)		2/363 (0.6)		1.24
Endocarditis	4/1065 (0.4)		1/359 (0.3)		1.69
Tracheotomy at discharge	116/1128 (10.3)				
Oxygen therapy at discharge	563/1128 (49.9)				

Analysis based on records from the COVID-19 Registry Japan. Data given as number of positive observations/total number of observations (percentage). For each variable, the number of missing observations can be obtained as the difference between the total number of patients in each phase and the total number of observations

For continuous variables, the standardized difference (*d*) is defined as follows:

d = ($$\overline{\chi }$$_survivor_ – $$\overline{\chi }$$_non-survivor_)/$$\sqrt{{(s}^{2}\mathrm{ survivor }+{s}^{2 }\mathrm{non}-\mathrm{survivor})/2}$$

For dichotomous variables, the standardized difference is defined as follows:

d = ($$\widehat{p}$$_survivor_ –$$\widehat{p}$$_non-survivor_)/$$\sqrt{\left\{\widehat{p} \mathrm{survivor }\left(1-\widehat{p} \mathrm{non}-\mathrm{survivor} \right)+\widehat{p} \mathrm{survivor }\left(1-\widehat{p} \mathrm{non}-\mathrm{survivor}\right)\right\}/2}$$

$\overline{\chi }$: mean, s: standard deviation, $\widehat{p}$: proportion

### Multivariable analysis for risk of in-hospital mortality

The multivariable analysis showed that a 10-year increase in age was significantly associated with mortality (Table 4). The odds ratio of death was 7 times higher in patients in their 70s (OR, 6.92; 95% confidence interval [CI] 4.23 to 11.31; *p* < 0.01), 13 times higher in patients in their 80s (OR, 13.17; 95% CI 7.21 to 24.06; *p* < 0.01), and 92 times higher in patients in their 90s (OR, 92.63; 95% CI 16.66 to 514.98; *p* < 0.01), compared with those aged < 60 years.

**Table 4:** Multiple logistic regression analysis of in-hospital mortality risk among coronavirus disease 2019 patients on mechanical ventilation after adjusting for within-hospital clustering

Variable	After multiple imputation		
	Odds ratio	95%CI	*p*-value
Age ≥ 90	92.63	16.66–514.98	< 0.01
80–89	13.17	7.21–24.06	< 0.01
70–79	6.92	4.23–11.31	< 0.01
60–69	2.60	1.65–4.08	< 0.01
59 ≤ (reference)	1		
Male	1.04	0.74–1.46	0.82
Body mass index ≥ 30 kg/m^2^	1.37	0.91–2.07	0.14
Smoking history	1.16	0.80–1.68	0.44
Comorbidity	1.33	0.87–2.03	0.19
Immunosuppression	2.17	1.14–4.12	0.02
Admission date			
3rd wave	0.86	0.59–1.26	0.45
2nd wave	0.62	0.41–0.95	0.03
1st wave (reference)	1		
Vital signs on admission			
Fever (≥ 38 °C)	0.83	0.61–1.13	0.25
SpO2 < 90%	1.00	0.68–1.46	0.99
Respiratory rate ≥ 30	1.02	0.70–1.50	0.90
Systolic blood pressure ≤ 80 mmHg	0.25	0.07–0.96	0.04
ARDS			
Severe	6.73	4.50–10.04	< 0.01
Moderate	0.63	0.39–1.02	0.06
Mild	0.54	0.23–1.28	0.16
None (reference)	1		
Drug administration for COVID-19	0.97	0.56–1.67	0.90
Steroid use	1.26	0.84–1.88	0.26
Drug administration for coagulopathy	0.96	0.68–1.35	0.82
Days from symptom onset to IMV	1.00	1.00–1.01	0.47

Analysis based on records from the COVID-19 Registry Japan

*ARDS* acute respiratory distress syndrome, *IMV* invasive mechanical ventilation

Severe ARDS was associated with high mortality rates (OR, 6.73; 95% CI 4.50 to 10.04; *p* < 0.01); however, moderate ARDS and mild ARDS were not related to mortality (OR, 0.63; 95% CI 0.39 to 1.02; *p* = 0.06, OR, 0.54; 95% CI 0.23 to 1.28; *p* = 0.16).

## Discussion

This nationwide cohort study assessed the relationship between mortality from COVID-19 and IMV, stratified by age. This study found that mortality drastically increased with increasing age among patients who required mechanical ventilation support.

The current study precisely reported mortality in COVID-19 patients who underwent IMV, which is the most critically ill group, in a large population. Although some studies have reported findings on critically ill patients with COVID-19, only a few large-sample surveys have focused on patients undergoing IMV, which is one of the most important treatment options for pneumonia and respiratory illness [4–7, 11, 12]. Therefore, this study may be valuable in understanding the epidemiology of severe respiratory dysfunction caused by COVID-19.

The results of our study demonstrated that increasing age was firmly associated with a higher risk of mortality in COVID-19 patients undergoing IMV. Although previous studies have reported the risk of advanced age, the current study suggested that age was associated with a higher risk in comparison to other factors, and that preventing progression to severe COVID-19 in elderly patients may be a great public health issue. Vaccination, careful observation for asymptomatic patients with COVID-19, and early treatment for symptomatic patients with COVID-19 may be strongly recommended for the people aged > 60 years.

This study also indicated other features of severe COVID-19. The definition of ARDS as a COVID-19 complication adopted in this study was based on the Berlin definition 2012; respiratory failure occurred within 1 week of known clinical insult or new or worsening respiratory symptoms (Additional file 1: Table S1) [15]. ARDS was not diagnosed in 44.3% of survivors and 25.7% of non-survivors, and the mean duration from symptom onset to IMV therapy was about 9 days in both groups. These results suggested that several patients struggling with severe COVID-19 showed gradual deterioration over a 1-week period, and required IMV therapy. However, multivariable analysis showed that severe ARDS was associated with high risk of mortality; that is, acute deterioration in COVID-19 patients might be a sign of worse outcome.

The strength of this study is its design, as it is a nationwide, multi-centre survey in Japan. Initially, we demonstrated some features of the Japanese medical system. As the Japanese health insurance system supports homogenizing and generalizing the Japanese medical system, the outcome of this study was the result of uniformed standard medical treatment, including IMV support, for all ages. In the present Japanese super-aging society, our study revealed that older age had a great effect on mortality associated with IMV therapy in COVID-19 patients, after adjusting for important variables that are independently associated with mortality. This result may be helpful in developing effective therapeutic strategies against COVID-19.

There are some limitations to the current study. First, Dawn et al., reported that high demand for the intensive care unit services and workload have an effect on mortality [24], and a similar situation was observed in Japan during the study period. In addition, the size of the hospitals that participated in the current study varied. Second, this database did not include information about the strain of COVID-19; therefore, we could not adjust for the effect of the COVID-19 strain on mortality. To reduce the effect of these two factors, we adjusted for hospital clustering and time course. Third, these results may not be generalizable to other countries where the medical and social systems are different from those in Japan. Fourth, the occurrence of the primary outcome might influence/preclude the occurrence of secondary outcomes (complications, tracheostomy, or oxygen therapy at discharge). However, we could not evaluate the cause of death in the current study. Thus, we could not evaluate the cause–effect relationship between the primary and secondary outcomes. Finally, the diagnosis of complications was made by each doctor in charge in the clinical setting. There might be a possibility of misdiagnosis because of these factors.

## Conclusion

The findings of this multi-centre, observational study, which assessed COVID-19 patients in Japan, demonstrated that age was a crucial prognostic factor in identifying patients at risk of dying among critically ill COVID-19 patients who required IMV. Further large-scale, prospective studies are required to validate our results.

## Supplementary Information

**Additional file 1: Table S1. **Definition of the variables for confounding factors using multivariable analysis in Table 4.

## Data Availability

The data that support the findings of this study are available from the corresponding author upon reasonable request.

## Abbreviations

COVID-19: Coronavirus disease 2019
IMV: Invasive mechanical ventilation
ARDS: Acute respiratory distress syndrome
BMI: Body mass index
COVIREGI-JP: COVID-19 Registry Japan
CI: Confidence interval

## References

[1] Zhu N, Zhang D, Wang W, Li X, Yang B, Song J, Zhao X, Huang B, Shi W, Lu R (2020). A novel coronavirus from patients with pneumonia in China, 2019. N Engl J Med.

[2] Coronavirus disease (COVID-19) outbreak, 2020. https://www.who.int/emergencies/diseases/novel-coronavirus-2019/events-as-they-happen.

[3] Current status of the novel coronavirus infection and the response of the MHLW. https://www.mhlw.go.jp/stf/nepage_/09290.html.

[4] Xie J, Wu W, Li S, Hu Y, Hu M, Li J, Yang Y, Huang T, Zheng K, Wang Y (2020). Clinical characteristics and outcomes of critically ill patients with novel coronavirus infectious disease (COVID-19) in China: a retrospective multicenter study. Intensive Care Med.

[5] Arentz M, Yim E, Klaff L, Lokhandwala S, Riedo FX, Chong M, Lee M (2020). Characteristics and outcomes of 21 critically ill patients with COVID-19 in Washington State. JAMA.

[6] Suleyman G, Fadel RA, Malette KM, Hammond C, Abdulla H, Entz A, Demertzis Z, Hanna Z, Failla A, Dagher C (2020). Clinical characteristics and morbidity associated with coronavirus disease 2019 in a series of patients in metropolitan detroit. JAMA Netw Open.

[7] Ioannou GN, Locke E, Green P, Berry K, O'Hare AM, Shah JA, Crothers K, Eastment MC, Dominitz JA, Fan VS (2020). Risk factors for hospitalization, mechanical ventilation, or death among 10131 US veterans with SARS-CoV-2 infection. JAMA Netw Open.

[8] Wu C, Chen X, Cai Y, Xia J, Zhou X, Xu S, Huang H, Zhang L, Zhou X, Du C (2020). Risk factors associated with acute respiratory distress syndrome and death in patients with coronavirus disease 2019 pneumonia in Wuhan, China. JAMA Intern Med.

[9] Richardson S, Hirsch JS, Narasimhan M, Crawford JM, McGinn T, Davidson KW, Barnaby DP, Becker LB, Chelico JD, the Northwell C-RC (2020). Presenting characteristics, comorbidities, and outcomes among 5700 patients hospitalized with COVID-19 in the New York City Area. JAMA.

[10] Rosenthal N, Cao Z, Gundrum J, Sianis J, Safo S (2020). Risk factors associated with in-hospital mortality in a US national sample of patients with COVID-19. JAMA Netw Open.

[11] Grasselli G, Greco M, Zanella A, Albano G, Antonelli M, Bellani G, Bonanomi E, Cabrini L, Carlesso E, Castelli G (2020). Risk factors associated with mortality among patients with covid-19 in intensive care units in Lombardy, Italy. JAMA Intern Med.

[12] Gupta S, Hayek SS, Wang W, Chan L, Mathews KS, Melamed ML, Brenner SK, Leonberg-Yoo A, Schenck EJ, Radbel J (2020). Factors associated with death in critically ill patients with coronavirus disease 2019 in the US. JAMA Intern Med.

[13] Nguyen NT, Chinn J, Nahmias J, Yuen S, Kirby KA, Hohmann S, Amin A (2021). Outcomes and mortality among adults hospitalized with COVID-19 at US Medical Centers. JAMA Netw Open.

[14] Matsunaga N, Hayakawa K, Terada M, Ohtsu H, Asai Y, Tsuzuki S, Suzuki S, Toyoda A, Suzuki K, Endo M (2020). Clinical epidemiology of hospitalized patients with COVID-19 in Japan: report of the COVID-19 REGISTRY JAPAN. Clin Infect Dis.

[15] Force ADT, Ranieri VM, Rubenfeld GD, Thompson BT, Ferguson ND, Caldwell E, Fan E, Camporota L, Slutsky AS (2012). Acute respiratory distress syndrome: the Berlin Definition. JAMA.

[16] Austin PC (2009). Balance diagnostics for comparing the distribution of baseline covariates between treatment groups in propensity-score matched samples. Stat Med.

[17] Little RJ, D’Agostino R, Cohen ML, Dickersin K, Emerson SS, Farrar JT, Frangakis C, Hogan JW, Molenberghs G, Murphy SA, Neaton JD (2012). The prevention and treatment of missing data in clinical trials. N Engl J Med.

[18] Janssen KJ, Donders AR, Harrell FE, Vergouwe Y, Chen Q, Grobbee DE, Moons KG (2010). Missing covariate data in medical research: to impute is better than to ignore. J Clin Epidemiol.

[19] Alhazzani W, Møller MH, Arabi YM, Loeb M, Gong MN, Fan E, Oczkowski S, Levy MM, Derde L, Dzierba A (2020). Surviving sepsis campaign: guidelines on the management of critically ill adults with coronavirus disease 2019 (COVID-19). Crit Care Med.

[20] Kazuma Y, Ryo Y, Go I, Hideki H, Takero T, Yoshitaka H. Japanese Rapid/Living recommendations on drug management for COVID-19. Acute Med Surg 2021, 2021(May 15). 10.1002/ams2.664 PMC8209876 34178358

[21] Steyerberg E (1999). Stepwise selection in small data sets a simulation study of bias in logistic regression analysis. J Clin Epidemiol.

[22] Steyerberg EW, Eijkemans MJC, Van Houwelingen JC, Lee KL, Habbema JDF (2000). Prognostic models based on literature and individual patient data in logistic regression analysis. Stat Med.

[23] Horby P, Lim WS, Emberson JR, Mafham M, Bell JL, Linsell L, Staplin N, Brightling C, Ustianowski A, Group RC (2021). Dexamethasone in Hospitalized Patients with Covid-19. N Engl J Med.

[24] Bravata DM, Perkins AJ, Myers LJ, Arling G, Zhang Y, Zillich AJ, Reese L, Dysangco A, Agarwal R, Myers J (2021). Association of intensive care unit patient load and demand with mortality rates in US department of veterans affairs hospitals during the COVID-19 pandemic. JAMA Netw Open.

