# Supplementary material for: Association between mortality and age among mechanically ventilated COVID-19 patients: a Japanese nationwide COVID-19 database study
Source: Ann Intensive Care. 2021 Dec 11;11:171. doi: 10.1186/s13613-021-00959-6 (PMC8665852; doi:10.1186/s13613-021-00959-6)
Supplement: Supplementary file 1 — Additional file 1: Table S1. Definition of the variables for confounding factors using multivariable analysis in Table 4. [file 13613_2021_959_MOESM1_ESM.docx]

**Table s1. Definition of the variables for confounding factors using multivariable analysis in table 4**

| Variable | Definition |
| --- | --- |
| Comorbidity | Ischemic heart disease, congestive heart failure, cerebrovascular disease, major neurocognitive disorder, COPD, chronic lung disease excluding COPD, bronchial asthma, liver disease, hypertension, hyperlipidemia, diabetes, moderate to severe kidney disease, haemodialysis before admission, solid tumor,  leukaemia, lymphoma, metastatic solid tumor, and collagen disease. |
| Immunosuppression | Neutropenia, use of steroid in the past 1 month, chemotherapy in the past 3 months, Radiotherapy in the past 3 months, Solid-organ transplantation and immunosuppressant use in the past 3 months. |
| Drug administration for COVID-19 | Favipravir, remdesivir, ciclesonide nafamostat, tocilizumab, hydroxychloroquine, Lopinavir and ritonavir, ivermectin, Interferon, and baricitinib |
| Drug administration for coagulopathy | Anticoagulant agents, antiplatelet agents, and thrombolytic agents. |
| Acute respiratory distress syndrome  (the Berlin definition 2012)[1] | Timing: within 1 week of known clinical insult or new or worsening respiratory symptoms  Chest imaging: bilateral opacities-not fully explained by effusions, lober/lung collapse, or nodules  Origin of edema: respiratory failure not fully explained by cardiac failure or fluid overload.  Oxygenation Mild: 200mmHg <PaO_2_/FiO2 ≤ 300mmHg with PEEP or CPAP ≥ 5 cm H_2_O  Moderate: 100mmHg <PaO_2_/FiO2 200mmHg with Peep ≥ 5 cm H_2_O  Severe: PaO_2_/FiO2 ≤ 100mmHg with Peep ≥5 cm H_2_O |

COPD, chronic obstructive pulmonary disease.

1. The ARDS definition task force: Acute respiratory distress syndrome. The Berlin definition. JAMA. 2012; 307:2526-2533.
